# The leaves of *Crataeva nurvala* Buch-Ham. modulate locomotor and anxiety behaviors possibly through GABAergic system

**DOI:** 10.1186/s12906-018-2338-y

**Published:** 2018-10-19

**Authors:** Md Moniruzzaman, Md Abdul Mannan, Md Farhad Hossen Khan, Ariful Basher Abir, Mirola Afroze

**Affiliations:** 1 Department of Pharmacy, Stamford University Bangladesh, 51 Siddeswari road, Dhaka, 1217 Bangladesh; 2 Designated Reference Institute for Chemical Measurement (DRiCM), Bangladesh Council of Scientific & Industrial Research, Dhaka, 1205 Bangladesh; 3 Mater Research Institute – UQ at Translational Research Institute, Faculty of Medicine, The University of Queensland, Brisbane, QLD 4102 Australia

**Keywords:** Capparidaceae, *Crataeva nurvala*, Sedative, Anxiolytic, Medicinal plant, GC/MS-MS

## Abstract

**Background:**

*Crataeva nurvala* Buch-Hum is an indigenous herb, extensively used in traditional medicines of the South Asian countries to treat inflammation, rheumatic fever, gastric irritation, and constipation. Despite this wide range of uses, very little information is known regarding its effects on the central nervous system (CNS). Therefore, this study evaluated the neuropharmacological properties of methanolic extract of *Crataeva nurvala* leaves (MECN) using a number of behavioral models in animals. This study also identified potentially active phytochemicals in MECN.

**Methods:**

Following MECN administration (at 50, 100 and 200 mg/kg; b.w.) the animals (male Swiss albino mice) were employed in hole-cross test (HCT), open field test (OFT), and rota-rod test (RRT) to evaluate sedative properties, where anxiolytic activities were investigated using elevated plus maze (EPM), light dark box (LDB), and marble burying test (MBT). The involvement of GABAergic system was evaluated using thiopental sodium (TS)-induced sleeping time determination test. Moreover, colorimetric phytochemical tests as well as GC/MS-MS were also conducted to define the phytochemical constituents of MECN.

**Results:**

MECN possesses sedative properties indicated through the dose-dependent inhibition of locomotor activities of the animals in HCT and OFT and motor coordination in RRT. MECN also exhibited prominent anxiolytic properties through decreased burying behavior in MBT, increased time spent and transitions in open arm of EPM, and increased time spent in light compartment of LDB. In addition, the treatments potentiated TS-mediated hypnosis indicating a possible participation of GABAergic system in the observed sedative and anxiolytic activities. Phytochemical screening of MECN revealed 48 different compounds in it. We reviewed and conceive that the sedative and anxiolytic effects could be due to the presence of neuroactive compounds such as phytol, D-allose, and α-Tocopherol in MECN.

**Conclusion:**

The present study showed that MECN possesses sedative and anxiolytic potential which could be beneficial in treatment of anxiety and insomnia associated with different psychological disorders.

## Background

Sleep disturbance and anxiety are the very common mental health problems world-wide, which have been regarded as underpinning factors to exacerbate different psychiatric diseases. Now-a-days the treatments for insomnia and anxiety consist of several synthetic and semisynthetic chemical compounds such as benzodiazepines, barbituric acids, and buspirone. However, these drugs have a number of undesirable side effects including amnesia, muscle relaxation, dependence, and tolerance [1, 2]. Therefore, searching for novel pharmacotherapy particularly from natural sources for neurological and psychiatric diseases has progressed significantly, owing to their better tolerability and fewer side effects. It is also evident that phytotherapies play a beneficial role for the patients who poorly respond to the conventional therapies [3, 4]. Therefore, the present study aimed to investigate *Crataeva nurvala* Buch. -Ham., a well-known member of Capparidaceae family, for its sedative and anxiolytic properties.

*C. nurvala* is a very common, medium-sized branched deciduous tree found in all over South Asian countries including Bangladesh, where the plant is known as Borun or Bonna [5]. Borun is extensively used in the traditional medicines due to its beneficial properties as memory enhancer, promoter of wound healing [6], laxative, lithotrophic, anti-inflammatory, contraceptive, febrifuge, and tonic. The traditional medicine practitioners also use this plant in treatment of kidney and bladder stone, vomiting, rheumatic fever, and gastric irritation [7]. Due to these diverse pharmacological properties, researchers have tried to validate the scientific basis of use of this plant. Their findings provided scientific evidence regarding the analgesic, antidiarrheal, and antinociceptive properties of the ethanol and methanol extracts of the leaves in mice, respectively [8]. On the other hand, Capparidaceae family particularly the genus *Crataeva* is almost unknown in their effects on the central nervous system. *Crataeva religiosa* is the only member reviewed to have stimulatory effects of autonomic nervous system [9]. Previously, we have shown that MECN has the potential to alleviate pain responses through controlling CNS, particularly the opioid system [8]. Therefore, we hypothesized that the chemical constituents of MECN might cross blood brain barrier and have capability to modulate brain functions directly. In 2014, Ali et al. and his colleagues reported the sedative effects of MECN using HCT, OFT, and EPM models with a single high dose (400 mg/kg) of the extract [10]. In our opinion, research outcomes from a single higher dose and a limited number of experimental models are unable to claim a specific pharmacological property, dose dependency, as well as mechanism of action of an agent. Moreover, the phytochemical constituents of MECN is still unknown.

Therefore, these limited study of the family Capparidaceae especially *C. nurvala* on the central nervous system (CNS) influenced us to design present study to investigate the sedative and anxiolytic properties of the extract using different behavioral models in mice.

## Methods

### Plant material and extraction

The leaves of *C. nurvala* were collected from Comilla, Bangladesh, in October 2012. The samples were then authenticated by Bushra Khan, Principal Scientific Officer of Bangladesh National Herbarium, Dhaka, Bangladesh. A voucher specimen (DACB: 37942) has been deposited in the Herbarium for further reference. 250 g of shed dried powdered leaves were macerated with 500 ml of methanol with occasional stirring for 72 h at 25 ± 2 °C temperature. The filtrate was then collected and made it dry using rotary evaporator and normal air flow resulting in 10.31 g extract (Yield 4.12%). This crude extract was further used for the acute toxicity, sedative, anxiolytic activity studies, and phytochemical analysis.

### Reagents

Diazepam and thiopental sodium were purchased from Square Pharmaceuticals Ltd. (Dhaka, Bangladesh). Methanol and tween 20 were procured from Merck (Darmstadt, Germany).

### Animals

Male *Swiss albino* mice of 20–25 g body weight were collected from icddr,b. Animals were housed in a standard environment maintaining 12 h light/dark cycle (7.00 am to 7.00 pm), 25 ± 2 °C room temperature, and 55–65% relative humidity. Flake wood shavings were used as bedding materials and icddr,b formulated standard diet and clean water ad libitum were provided in their regular meal. Prior to the experiments, 14 days of acclimatization period was maintained. All experimental animals were treated following the Health guide for the care and use of Laboratory animals (1978) formulated by the National Institute of Health. All protocols conducted in this study comply with the ARRIVE guidelines and were approved by the Institutional Animal Ethics Committee of Stamford University Bangladesh (SUB/IAEC/14.09). Moreover, pentobarbital was used to euthanize the animals following AVMA guidelines for the Euthanasia of Animals (2013).

### Drugs and treatments

Mice were divided into 5 different groups containing 5 animals in each. In all experiments, diazepam at 1 mg/kg was used as a reference standard and administered through intraperitoneal (i.p.) route 15 min before the experiments. On the other hand, MECN was dissolved in 0.2% tween 20 and orally administered to the animals at the doses of 50, 100 and 200 mg/kg body weight (adjusted volume 0.1 ml/mouse). Animals from the control group only received 0.2% tween 20 (vehicle; p.o.) 30 min prior to the experiments. Moreover, thiopental sodium (20 mg/kg; i.p.) in sleeping time measurement test was injected 15 min after MECN or diazepam treatments.

### Acute toxicity test

The animals were divided into five consecutive groups containing five animals in each. Animals were kept in close observation for 72 h and in a total of seven days following the oral treatments with vehicle or MECN at 500, 1000, 2000, and 3000 mg/kg doses (adjusted volume 0.1 ml/mouse) to check any allergic reaction, swelling, vomiting, diarrhea, and mortality induced by MECN. In the meantime they were allowed to have access to food and water ad libitum [11].

### Sedative activity analysis

#### Hole cross test

The hole cross box is a cage of (30 × 20 × 14) cm^3^ in size with a partition in the middle having a hole of 3 cm in diameter. Mice were treated with vehicle, MECN or diazepam and placed in one chamber of the cage. Then the total number of passages of a mouse through the hole from one compartment to another was counted for a period of 3 min before and after 30, 60, 90 and 120 min of the treatments [12]. The percentage of inhibition was calculated for each time point according to the following formula:

$$ \%\mathrm{Inhibition}=\left[\left(\mathrm{Control}-\mathrm{Treatment}\right)/\mathrm{Control}\right]\times 100 $$

#### Open field test

This test is one of the most frequently used methods to evaluate locomotor activity and emotionality of the rodents. The apparatus is a square box consisting of a 50 cm high wall and a wooden floor with a series of squares alternatively painted in black and white. Animals were administered with vehicle, MECN, or diazepam and placed in the middle of the open field allowing free exploration. The animals were then scored with the number of squares they visited for 3 min before and at 30, 60, 90 and 120 min post treatments [13]. The percentage of inhibition was calculated for each time point as described in hole cross test.

#### Test for motor co-ordination (Rota-rod test)

The apparatus consists of a non-slippery plastic rod of 3 cm in diameter (Ugo Basile, Varese, Italy) which rotates at the speed of 20 rpm. The animals which were able to stay in the rotating rod for more than 180 s were selected for the study. Animals were then treated with vehicle or MECN or diazepam and placed on the rotating rod to register their falling latency from the rod within 180 s [14, 15]. The percentage of inhibitions were calculated as described in hole cross test.

### Anxiolytic activity analysis

#### Elevated plus-maze test (EPM)

The plus-maze apparatus is consisting of two open arms (15 × 5 cm^2^) and two closed arms (15 × 5 × 5 cm^3^) extending from a central platform (5 × 5 cm^2^), and raised 50 cm above the floor level. Animals were randomly selected for each group and treated with vehicle, MECN, or drug. Then each animal was placed at the center of the plus-maze and allowed them to freely access the maze for 3 min. The number of entries and total time spent in open arms were then recorded within the indicated time [16].

#### Light-dark box exploration test (LDB)

The apparatus is an open-topped rectangular box (46 × 27 × 30 cm^3^), divided into a small (18 × 27 cm^2^) and a large (27 × 27 cm^2^) compartments with a fixed partition containing a small hole (3 cm in diameter) in the middle. The small compartment was closed with a lid, painted black and illuminated with a dim light. On the other hand, the large compartment was painted in white and a 60 W electric bulb was hanged on at the top (120 cm above) to brightly illuminate it. Mice were treated with vehicle, MECN, or diazepam and placed in the middle of the open compartment. Then the time spent by the animals in open compartment and total number of transitions between the compartments were recorded for 3 min [17, 18].

#### Marble burying test (MBT)

A normal glass cage with bedding materials was used in this experiment. Before testing, individual animal was acclimatized in one cage for 30 min. After removal of the animal, 25 glass marbles were uniformly distributed on top of the 4 cm layer of bedding materials. Following MECN, vehicle, or diazepam treatment, each animal was replaced in the cage for 30 min. The number of buried marbles were then counted as a score of anxiety [19]. The percentage of inhibitions were calculated as described in hole cross test.

### Thiopental sodium-induced sleeping time test

The animals were randomly divided into desired groups and administered with vehicle, MECN, or diazepam. Thirty min after vehicle or MECN and 15 min after standard drug, thiopental sodium (TS) was administered to each animal i.p. at a dose of 20 mg/kg. Then animals were observed for the latent period (time between TS administration and loss of their righting reflex) and the duration of sleep (time between the loss and recovery of righting reflex)-induced by TS [14].

### Phytochemical analysis

MECN was qualitatively evaluated to detect the presence of alkaloids, carbohydrates, glycosides, flavonoids, tannins, and reducing sugars according to the protocols described by Ghani et al. [20].

### GC/MS-MS analysis

GC/MS-MS analysis of the methanolic extract of the leaves of *C. nurvala* was carried out using GCMS-TQ8040 gas chromatograph mass spectrometer (Shimadzu Corp., Kyoto, Kyoto Prefecture, Japan) with a Rxi-5 ms fused silica capillary column (30 m × 0.25 mm × 0.25 μm film thickness). For GC, the injection temperature was set at 250 °C. The oven temperature was programmed at 50 °C for 1 min, 25 °C/min to 125 °C for 0 min, and 10 °C/min to 300 °C for 10 min. Total analysis time was 31.50 min where the column flow rate was 1.69 ml/min Helium gas. The MS was electron ionization (EI) type and set in Q3 scan mode. The ion source temperature was maintained at 200 °C and the interface at 250 °C. The detector voltage was set at 0.2 kV and the mass range was 50–1000 m/z. The individual compound was searched and identified using “NIST-MS Library 2009”. Total Ionic Chromatogram (TIC) was used to determine the peak area as well as the percentage amounts of each compound.

### Statistical analyses

The results are presented as Mean ± SEM. The statistical analysis was performed using one-way analysis of variance (ANOVA) followed by Dunnett’s post hoc test, except for the HCT and OFT. For these tests, two-way ANOVA followed by Bonferroni’s post hoc tests was adopted. In all the cases *P* < 0.05 was considered as significant. All statistical analysis was performed using SPSS software. Moreover, the pED_50_ and Hill Slope values were calculated using Graphpad Prism software.

## Results

### Hole cross and open field tests

Our results demonstrated that MECN significantly decreased the spontaneous locomotor activity in mice which is evident in both HCT (*F*_4,100_ = 90.67, *p* < 0.001) and OFT (*F*_4,100_ = 232.9, *p* < 0.001) models. The suppressive effect was observed at 30 min after oral administration with all MECN doses (50, 100 and 200 mg/kg) and continued up to 120 min of the observation period. However, the major drawbacks of these models are the animals get habituated and lost their curiosity due to repeated exposure in the same environment, which result in a decrease in their ambulatory activities. As expected, we observed the same results with animals from the control group. However, the decrements with MECN doses are higher and dose-dependent. As the locomotor activity of the animals has been decreased with time and doses, we performed two-way ANOVA analysis followed by Bonferroni’s post hoc test and found the effects of MECN are significantly (*P* < 0.05) different from the control group (Tables 1 and 2).

**Table 1:** Effect of MECN on hole cross test

Treatment	Dose (mg/kg)	Number of hole crossed (% of Inhibition)				
		Pretreatment	30 min	60 min	90 min	120 min
Control	0.1 ml/mouse	23.40 ± 1.21	18.60 ± 1.44	18.20 ± 1.36	17.00 ± 1.22	15.60 ± 1.08
Diazepam	1	21.40 ± 2.32	6.20 ± 1.07*** (66.67)	3.80 ± 0.58*** (79.12)	2.60 ± 0.51*** (84.71)	1.40 ± 0.24*** (91.03)
MECN	50	22.80 ± 1.28	15.40 ± 0.93 (17.20)	11.80 ± 0.73*** (35.16)	7.80 ± 0.86*** (54.12)	6.00 ± 0.71*** (61.54)
MECN	100	20.60 ± 1.86	10.60 ± 0.75*** (43.01)	9.00 ± 0.71*** (50.55)	4.60 ± 0.93*** (72.94)	3.80 ± 0.86*** (75.64)
MECN	200	21.20 ± 1.85	8.00 ± 0.71*** (56.99)	5.20 ± 0.73*** (71.43)	2.40 ± 0.51*** (85.88)	1.60 ± 0.24*** (89.74)

Effect of MECN on hole cross test. Values are presented as the Mean ± SEM (*n* = 5). MECN = Methanolic extract of *Crataeva nurvala*; ***p < 0.001 compared with the control group (two-way ANOVA followed by Bonferroni’s test)

**Table 2:** Effect of MECN on open field test

Treatment	Dose (mg/kg)	Number of square crossed (% of Inhibition)				
		Pretreatment	30 min	60 min	90 min	120 min
Control	0.1 ml/mouse	103.80 ± 3.51	94.40 ± 4.31	83.20 ± 3.09	74.60 ± 3.20	66.80 ± 2.58
Diazepam	1	101.60 ± 3.89	49.20 ± 2.13*** (47.88)	33.20 ± 1.88*** (60.10)	14.80 ± 1.28*** (80.16)	5.40 ± 1.12*** (91.92)
MECN	50	98.00 ± 2.83	67.40 ± 3.79*** (28.60)	42.00 ± 2.49*** (49.52)	31.60 ± 2.87*** (57.64)	16.20 ± 2.40*** (75.75)
MECN	100	100.80 ± 2.62	46.40 ± 2.75*** (50.85)	35.80 ± 2.48*** (56.97)	16.20 ± 4.53*** (78.28)	5.60 ± 0.75*** (91.62)
MECN	200	102.20 ± 3.44	34.40 ± 3.23*** (63.56)	22.20 ± 3.88*** (73.32)	10.80 ± 2.89*** (85.52)	4.20 ± 0.58*** (93.71)

Effect of MECN on open field test. Values are presented as the Mean ± SEM (*n* = 5). MECN = Methanolic extract of *Crataeva nurvala*; ***p < 0.001 compared with the control group (two-way ANOVA followed by Bonferroni’s test)

### Test for motor co-ordination

**Fig. 1:**
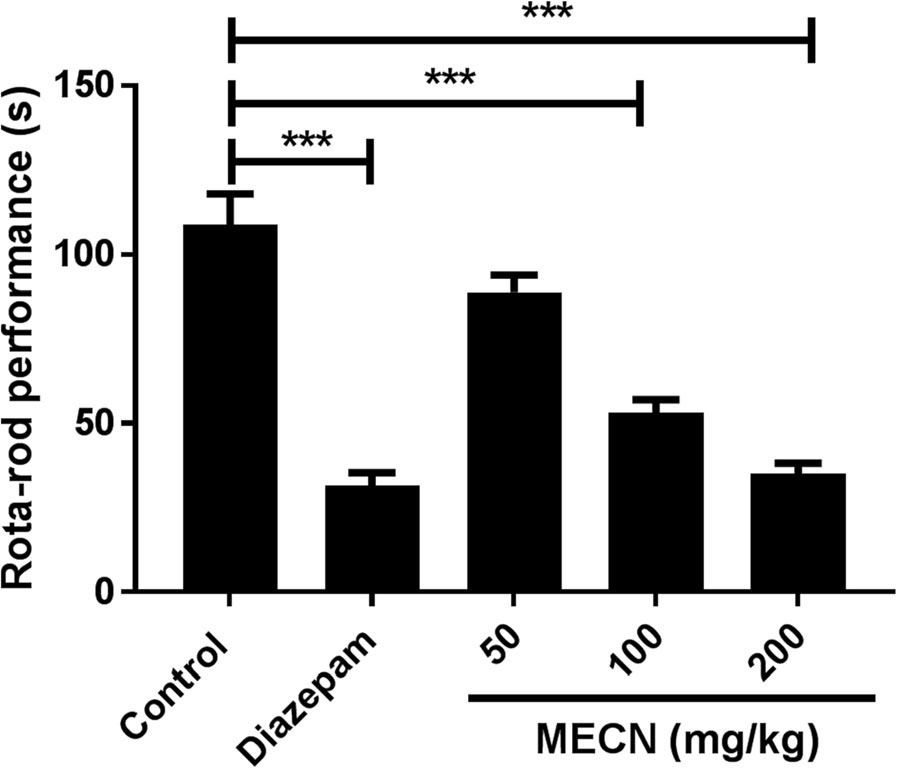
Rota-rod performance of mice. Animals were administered with MECN and exposed to the rota-rod to record their falling latency as described in the methods. Data are presented as Mean ± SEM (*n* = 5). pED_50_ value is 2.05 with the Hill slope of 1.55. MECN = Methanolic extract of *Crataeva nurvala*; *p < 0.05; **p < 0.01; ***p < 0.001 compared with the control group (one-way ANOVA followed by Dunnett's test)

As depicted in Fig. 1, the results showed that acute oral administration with MECN (50, 100 and 200 mg/kg doses) decreased the time that the mice were able to stay on the rota-rod. The highest performance inhibitions were observed with diazepam (71.01%) and MECN at 200 mg/kg (67.76%). Although, MECN dose-dependently suppressed motor co-ordination of the animals, the significant (*F*_4,20_ = 39.13, *p* < 0.001) inhibition was found with only higher doses of the extract (100 and 200 mg/kg). The estimated pED_50_ value is 2.05 with the Hill slope of 1.55.

### Elevated plus-maze test

**Fig. 2:**
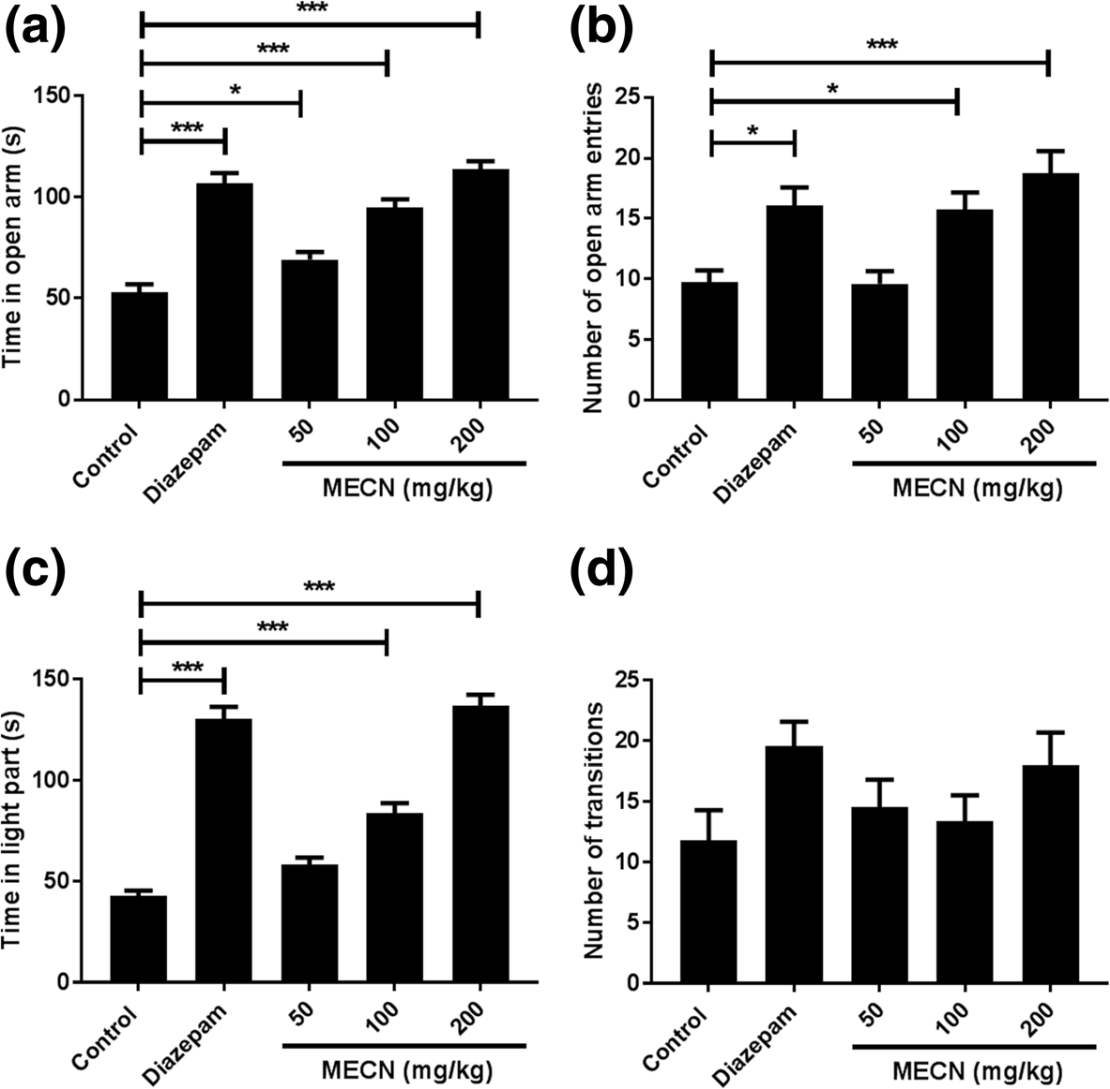
Effects of MECN on elevated plus maze (EPM) and light-dark box (LDB) tests. Following 30 min of drugs administration, animals were placed in the EPM to record the time they spent (pED_50_: 1.81; Hill slope: 3.55) (**a**) as well as number of transitions (pED_50_: 1.97; Hill slope: 6.71) (**b**) in the open arm. In LDB, mice were observed for the time spent (pED_50_: 1.74; Hill slope: 6.65) (**c**) and total number of transitions (pED_50_: 2.33; Hill slope: 1.35) (**d**) in the open lighted compartment. Data are presented as Mean ± SEM (n = 5). MECN = Methanolic extract of *Crataeva nurvala*; *p < 0.05; **p < 0.01; ***p < 0.001 compared with the control group (one-way ANOVA followed by Dunnett's test)

In EPM test, as expected for a positive control, diazepam at 1 mg/kg produced a selective anxiolytic effect in mice characterized by a significant (*F*_4,20_ = 36.28, *p* < 0.001) increase in the time spent as well as the number of entries in the open arm of EPM. Besides, treatments with the methanolic extract of *C. nurvala* at 100 and 200 mg/kg doses significantly (*F*_4,20_ = 36.28, *p* < 0.001) increased the spending time (pED_50_: 1.81; Hill slope: 3.55) and total number of entries (*F*_4,20_ = 8.50, *p* < 0.001; pED_50_: 1.97; Hill slope: 6.71) in the open arm (Fig. 2a, b).

### Light-dark box exploration test

The results obtained in this test demonstrated a significant (*F*_4,20_ = 78.56, *p* < 0.001) increase in time spent in LDB light chamber by MECN (pED_50_: 1.74; Hill slope: 6.65). It also increased the number of transitions between compartments in a dose-dependent manner (pED_50_: 2.33; Hill slope: 1.35) (Fig. 2c, d).

### Marble burying test

In this study, the oral administration of MECN at 50, 100 and 200 mg/kg doses showed dose-dependent and significant (*F*_4,20_ = 6.60, *p* < 0.01) inhibition of burying behavior of the animals (Table 3). The calculated pED_50_ value for MBT is 1.96 with the Hill slope of 2.09. Therefore, keeping with the results obtained in EPM and LDB tests, these findings support the anxiolytic potential of MECN.

**Table 3:** Effect of MECN on marble burying test

Treatment	Dose (mg/kg)	Responses	
		Numbers of marbles buried	% Inhibition
Control	0.1 ml/mouse	11.80 ± 2.52	0
Diazepam	1	1.20 ± 0.20**	89.83
MECN	50	9.40 ± 2.23	20.34
MECN	100	5.00 ± 2.10*	57.63
MECN	200	2.20 ± 0.58**	81.36

Effect of MECN on marble burying test. Values are presented as the Mean ± SEM (*n* = 5). MECN = Methanolic extract of *Crataeva nurvala*; *p < 0.05; **p < 0.01; compared with the control group (one-way ANOVA followed by Dunnett’s test)

### Thiopental sodium-induced sleeping time test

Our results demonstrated that the acute oral administration of MECN at 50, 100 and 200 mg/kg reduced the TS-mediated induction of sleep in the animals. However, the significant (*F*_4,20_ = 8.31, *p* < 0.01) effects were observed with the higher doses of MECN (100 and 200 mg/kg) for sleeping onset. These result in prolonged duration of sleep (*F*_4,20_ = 46.14, *p* < 0.001; pED_50_: 2.07; Hill slope: 3.50) as summarized in Table 4.

**Table 4:** Effect of MECN on thiopental sodium induced hypnosis

Treatment	Dose (mg/kg)	Responses	
		Onset of sleeping	Sleeping duration
Control	0.1 ml/mouse	9.24 ± 0.25	50.20 ± 1.86
Diazepam	1	6.97 ± 0.19***	98.40 ± 3.83***
MECN	50	8.87 ± 0.60	49.00 ± 4.85
MECN	100	7.92 ± 0.27*	69.60 ± 3.12*
MECN	200	7.18 ± 0.28**	92.20 ± 2.44***

Effect of MECN on thiopental sodium induced hypnosis. Values are presented as the Mean ± SEM (*n* = 5). MECN = Methanolic extract of *Crataeva nurvala*; *p < 0.05; **p < 0.01; ***p < 0.001 compared with the control group (one-way ANOVA followed by Dunnett’s test)

### Acute toxicity test

The acute toxicity test revealed that the oral administration of MECN at the doses of 500, 1000 2000, and 3000 mg/kg did not show any mortality or allergic manifestations during 7 days of the observation period. Therefore, it can be assumed that MECN possesses low toxicity profile and safe within our experimental doses up to the 3000 mg/kg.

### Phytochemical analysis

The preliminary phytochemical analysis revealed the presence of alkaloids, glycosides, carbohydrates, flavonoids, and tannins in MECN (Table 5).

**Table 5:** Groups of phytochemicals identified in MECN

Phytochemicals	Names of the tests	Expected changes	Results
Alkaloids	Mayer’s test	Yellowish buff color precipitate	+
	Hager’s test	Yellow crystalline precipitate	–
	Wagner’s test	Brown or deep brown precipitate	+
	Dragendorff’s test	Orange or orange-brown precipitate	+
	Tannic acid test	Buff color precipitate	+
Tannins	Ferric chloride test	Blue green color	+
	Alkaline reagent test	Yellow to red precipitate	+
Glycosides	General test	Yellow color	+
	Test for glucoside	Production of brick-red precipitation	+
Carbohydrates	Molisch’s test	A red or reddish violet ring is formed at the junction of two layers, and on shaking a dark purple solution is formed	+
	Barfoed’s test (general test for monosaccharides)	Red precipitate	+
	Fehling’s test	A red or brick-red precipitate	+
	Test for reducing sugar	A brick-red precipitate	+
Flavonoids	Hydrochloric acid reduction test	Red color	+

### GC/MS-MS analysis

**Fig. 3:**
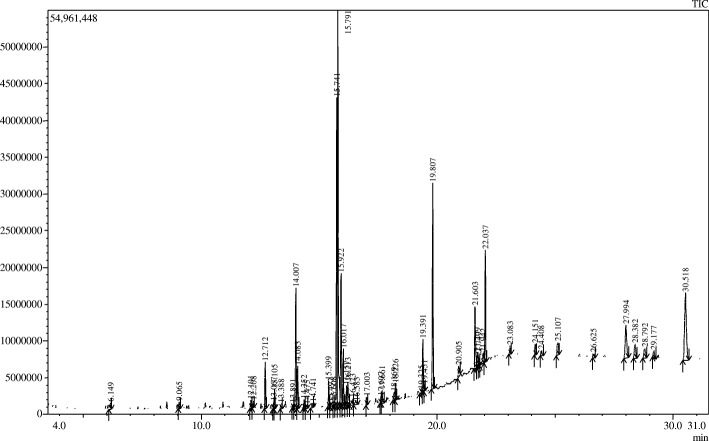
Total ionic chromatogram of MECN from GC/MS-MS

The GC/MS-MS analysis of MECN confirmed the presence of 48 different compounds (Fig. 3, Table 6). The major 25 compounds identified with % peak area are γ-Sitostenone (19.44%), Phytol (14.84%), 13-Docosenamide, (Z)- (8.73%), (S,E)-4-Hydroxy-3,5,5-trimethyl-4-(3-oxobut-1-en-1-yl)cyclohex-2-enone (5.24%), 7,9-Di-tert-butyl-1-oxaspiro(4,5)deca-6,9-diene-2,8-dione (4.36%), Ethanol, 2,2′-(dodecylimino)bis- (4.07%), 4-Campestene-3-one (3.49%), β-Amyrone (3.40%) Vitamin E (3.23%), Lup-20(29)-en-3-one (2.30%), Neophytadiene (1.98%), γ-Tocopherol (1.86%), (E)-4-(3-Hydroxyprop-1-en-1-yl)-2-methoxyphenol (1.28%), α-Tocospiro B (1.28%), Cyclopentane, (4-octyldodecyl)- (1.16%), 4,22-Cholestadien-3-one (1.08%), 6-Hydroxy-4,4,7a-trimethyl-5,6,7,7a-tetrahydrobenzofuran-2(4H)-one (1.05%), Bicyclo[4.1.0]heptan-3-one, 4,7,7-trimethyl-, [1R-(1.alpha.,4.beta.,6.alpha.)]- (1.02%), 2,3a-Dimethylhexahydrobenzofuran-7a-ol (1.02%), 2-Methylhexacosane (1.02%), Megastigmatrienone (0.96%), N,N-Diethylhexylamine (0.93%), Ergost-5-en-3-ol, (3.beta.)- (0.93%), Cholesta-4,6-dien-3-one (0.93%), and D-Allose (0.84%).

**Table 6:** Phytochemicals detected in MECN using GC/MS-MS

SN	RT (min)	% PA	Compound name	Biological activity	References
1	6.04	0.55	Catechol	Antioxidant	[39]
2	6.15	0.64	Butanenitrile, 3-chloro-3-methyl-	Not found	–
3	8.98	0.44	n-Tridecan-1-ol	Anti-alcoholic	[40]
4	9.07	0.84	D-Allose	Hepatoprotective, immunosuppressant, antioxidant, and anticancer.	[41–44]
5	10.92	0.96	Megastigmatrienone	Not found	–
6	11.1	0.70	2-Cyclohexen-1-one, 4-(3-hydroxy-1-butenyl)-3,5,5-trimethyl-	Not found	–
7	11.78	0.90	Ethanol, 2-(dodecyloxy)-	Antithyroid, supress varicose veins formation.	[45, 46]
8	11.96	0.44	Cedrol	Prevent chemotherapy-induced alopecia, promote hair growth, anticancer.	[47–49]
9	12.1	1.28	(E)-4-(3-Hydroxyprop-1-en-1-yl)-2-methoxyphenol	Not found	–
10	12.21	1.05	6-Hydroxy-4,4,7a-trimethyl-5,6,7,7a-tetrahydrobenzofuran-2(4H)-one	Not found	–
11	12.4	0.35	5,5,8a-Trimethyl-3,5,6,7,8,8a-hexahydro-2H-chromene	Not found	–
12	12.71	5.24	(S,E)-4-Hydroxy-3,5,5-trimethyl-4-(3-oxobut-1-en-1-yl)cyclohex-2-enone	Anticholinesterase and antioxidant	[50]
13	12.94	0.44	1(2H)-Naphthalenone, 3,4,5,6,7,8-hexahydro-	Not found	–
14	13.07	0.93	N,N-Diethylhexylamine	Not found	–
15	13.11	1.98	Neophytadiene	Not found	–
16	13.18	0.58	Z-28-Heptatriaconten-2-one	Not found	–
17	13.39	1.02	Bicyclo[4.1.0]heptan-3-one, 4,7,7-trimethyl-, [1R-(1.alpha.,4.beta.,6.alpha.)]-	Not found	–
18	13.392	1.02	2,3a-Dimethylhexahydrobenzofuran-7a-ol	Not found	–
19	13.89	0.73	1-(3-Hydroxymethyl-phenyl)-heptan-1-ol	Not found	–
20	14.09	4.36	7,9-Di-tert-butyl-1-oxaspiro(4,5)deca-6,9-diene-2,8-dione	Not found	–
21	14.47	0.81	9-(3,3-Dimethyloxiran-2-yl)-2,7-dimethylnona-2,6-dien-1-ol	Not found	–
22	14.48	0.84	1,4-Dimethyladamantane	Not found	–
23	14.74	0.49	Diethylene glycol monododecyl ether	Not found	–
24	14.79	0.61	trans-Sinapyl alcohol	Not found	–
25	15.92	14.84	Phytol	Anxiolytic, antitubercular and anticancer	[36, 51, 52]
26	16.21	4.07	Ethanol, 2,2′-(dodecylimino)bis-	Not found	–
27	16.69	0.52	Hexadecane	Not found	–
28	18.37	0.55	(2,2,6-Trimethyl-bicyclo[4.1.0]hept-1-yl)-methanol	Not found	–
29	18.79	0.61	7-Hexadecenal, (Z)-	Pheromone	[53]
30	19.32	1.02	2-Methylhexacosane	Not found	–
31	19.92	0.73	Ethyl 13-docosenoate(ethyl erucate)	Not found	–
32	20.13	0.58	Hexacontane	Not found	–
33	20.39	0.47	7-Methyl-6-oxo-1,2,3,4-tetrahydro-6H-pyrimido[1,2-a]pyrimidine	Not found	–
34	21.43	1.16	Cyclopentane, (4-octyldodecyl)-	Not found	–
35	21.6	8.73	13-Docosenamide, (Z)-	Not found	–
36	22.29	0.52	α-Tocospiro A	Not found	–
37	22.49	1.28	α-Tocospiro B	Not found	–
38	24.15	1.86	.gamma.-Tocopherol	Anti-inflammatory	[54, 55]
39	25.11	3.23	Vitamin E	Antioxidant	[56]
40	26.63	0.93	Ergost-5-en-3-ol, (3.beta.)-	Not found	–
41	27.08	0.49	Stigmasterol	Anti-asthmatic, anti-inflammatory, anti-proliferative, anti-bacterial, acetylcholinesterase inhibitor	[57–60]
42	28.38	3.40	β-Amyrone	Not found	–
43	28.79	3.49	4-Campestene-3-one	Not found	–
44	28.96	0.55	Cholestan-3-one, (5.alpha.)-	Not found	–
45	29.18	2.30	Lup-20(29)-en-3-one	Melanogenesis, hypolipidemic, anti-inflammatory, antidiabetic	[61–64]
46	29.35	1.08	4,22-Cholestadien-3-one	Not found	–
47	30.52	19.44	γ-Sitostenone	Not found	–
48	31.14	0.93	Cholesta-4,6-dien-3-one	Not found	–

*SN* serial number, *RT* retention time, *PA* peak area

## Discussion

**Fig. 4:**
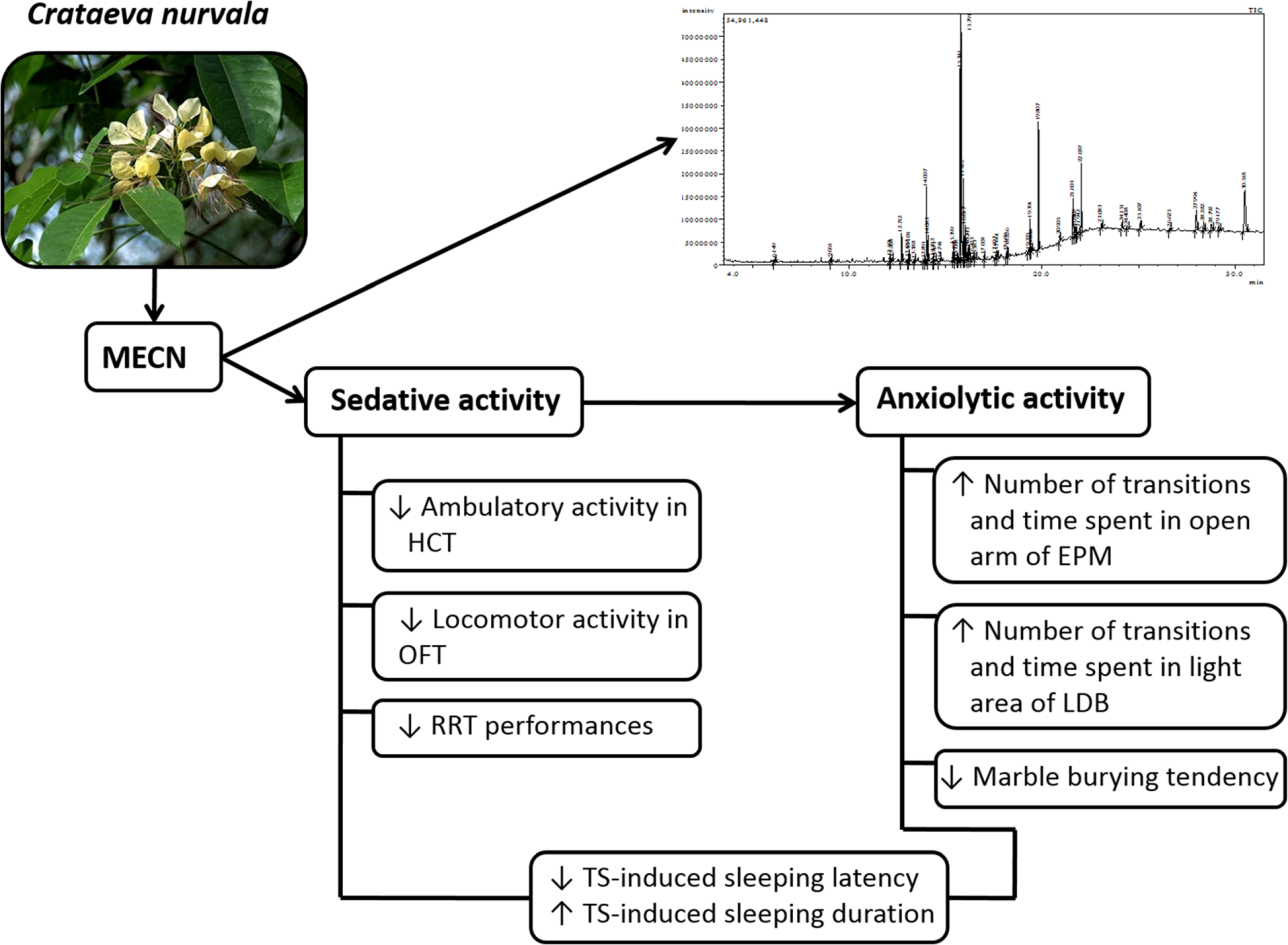
Sedative and anxiolytic activities of MECN

The present study was carried out to investigate the effects of MECN on the central nervous system (Fig. 4), which is the first extensive study reporting the psychoactive potential of a member of the genus *Crataeva*. In hole cross and open field tests, the oral administration of MECN significantly suppressed spontaneous locomotor behavior of the animals indicating that MECN possesses at least some sedative properties. These observations were supported by a previously reported work, where the locomotor activity of the animals was suppressed by MECN at 400 mg/kg dose [10]. Moreover, our earlier study revealed that MECN involves opioid system to show its antinociceptive effect in mice which was confirmed by using a non-specific opioid receptors antagonist, naloxone [8]. It has been reported that the opioid system inhibits GABAergic interneurons and directly affect the dopaminergic system to control locomotor behaviors [21]. Therefore, in agreement with these reports, it is conceivable that MECN may also involve opioid pathway to exhibit its sedative activity.

Motor coordination is a complex behavioral domain that reflects muscle strength, balance, and patterned gait [22]. It has been reported that barbiturates, benzodiazepines, and similar compounds can cause muscle weakness [23], sedation, and decrease ambulatory activity resulting in an impaired performance in rota-rod [24]. In our study, the administration of MECN and standard drug diazepam parallelly impaired motor co-ordination in rota-rod test, indicating that MECN has the capability to produce sedation that affects general activity and motor coordination of the mice.

To investigate the anxiolytic effect of MECN, we used EPM, a widely used animal model to scrutinize anxiolytic drugs. This experimental model acts based on the innate aversion of mice/rats to open place. In this test, the number of entries and total time spent in the open arms have generally been used as the indication of anxiolytic effect [25]. Like EPM, LDB is also a classical model of screening anxiolytic or anxiogenic drugs. LDB works based on the inherent unwillingness of rodents to enter into the brightly illuminated areas and on their spontaneous exploratory behaviors in response to mild stressors like light and novel environment. It has been reported that not the number of transitions but the time spent in the light area is the most consistent and useful parameter in investigation of anxiolytic action [26]. We found that MECN treatments influenced the animals to come in open and lighted areas of EPM and LDB, disclosing the anxiolytic potential of MECN. Moreover, the glass marbles in marble burying test provide an effective unconditioned stimulus which incites burying. This aversive stimulus has been reported sensitive to diazepam or chlordiazepoxide and suggested that the decrease in burying behavior will indicate the anxiolytic action of a drug candidate [19, 27]. Therefore, our findings in MBT suggest the prominent anxiolytic action of MECN which is consistent with the results obtained from EPM and LDB tests.

To confirm the possible involvement of GABAergic system in MECN-mediated sedative and anxiolytic activity, we co-administered thiopental sodium (TS) with MECN or diazepam. It is well established that the sedative and anxiolytic effect of benzodiazepines are due to their binding to GABA_A_ receptor at a site distinct from the GABA binding site [28]. However, when barbiturates like TS binds with the barbituric acid binding sites of GABA_A_ receptor complex, it potentiates GABA-mediated hyperpolarization of postsynaptic neurons [29]. Therefore, it has been suggested that the agents including natural products and chemical compounds which potentiate thiopental-induced sleeping, will potentially affect GABAergic neurotransmission [30, 31]. In corroboration with our results it is therefore conceivable that GABAergic system might have the contribution at least in a part in MECN-induced modulation of the sleeping behavior of animals produced by TS.

The qualitative phytochemical analysis identified the presence of alkaloids, glycosides, carbohydrates, flavonoids, and tannins in MECN. Although GC is mainly effective for identification of volatile compounds, we further analyzed MECN using GC/MS-MS due to the instrument availability and accessibility. Among others, D-Allose is the most active compound in MECN, reported to inhibit mitochondrial reactive oxygen species in Neuro2A cells [32] as well as protect the brain from transient ischemic neural death [33]. During cerebral ischemia, this compound also inhibited the production of inflammatory cytokines and translocation of NF-κB components to protect blood-brain barrier [34, 35]. Phytol was found to produce significant anxiolytic activity in mice which is mediated through its interaction with GABAergic system [36]. Moreover, it has also been reported that α-Tocopherol (vitamin E) and other alkaloids, flavonoids, tannins, and terpenoids containing plant extracts possess strong sedative and anxiolytic and anticonvulsant effects on Swiss albino mice [37, 38]. Although the direct involvements of individual components are yet to be revealed, accumulated evidence raise the possibility of these reported neuro-active components to participate at least in a part in the mechanism of sedative and anxiolytic actions of MECN.

## Conclusion

Taken together, we conclude that MECN possesses strong sedative-and anxiolytic-like properties which may be mediated through the interactions with GABAergic system that affect the general activity, motor coordination and muscle strength of the animals. We also hypothesize that the observed effects are due to the presence of several psychoactive phytochemicals including phytol, D-allose, and α-tocopherol in MECN. Therefore, further in-depth studies are required to isolate the bioactive phytochemical(s) and understand the precise molecular mechanisms underlying the observed pharmacological activities. In addition, we also believe that this work will act as an eye-opener regarding the central nervous system effect of the genus *Crataeva* and will serve as a basis for future research of MECN.

## Abbreviations

CNS: Central nervous system
EPM: Elevated plus maze
GC/MS-MS: Gas chromatography-mass spectroscopy-mass spectroscopy
HCT: Hole cross test
icddr,b: International Center for Diarrheal Disease and Research, Bangladesh
LDB: Light-dark box
MBT: Marble burying test
MECN: Methanol extract of *C. nurvala* leaves
OFT: Open field test
RRT: Rota-rod test
TS: Thiopental sodium
